# Hyperspectral imaging for the detection of plant pathogens in seeds: recent developments and challenges

**DOI:** 10.3389/fpls.2024.1387925

**Published:** 2024-04-11

**Authors:** Luciellen da Costa Ferreira, Ian Carlos Bispo Carvalho, Lúcio André de Castro Jorge, Alice Maria Quezado-Duval, Maurício Rossato

**Affiliations:** ^1^ University of Brasilia, Departament of Plant Pathology, Brasília, Brazil; ^2^ Embrapa Instrumentação, São Carlos, Brazil; ^3^ Embrapa Hortaliças, Brasília, Brazil

**Keywords:** artificial intelligence, phytopathogen, machine learning, seedborne, bacteria, fungus, virus, nematode

## Abstract

Food security, a critical concern amid global population growth, faces challenges in sustainable agricultural production due to significant yield losses caused by plant diseases, with a multitude of them caused by seedborne plant pathogen. With the expansion of the international seed market with global movement of this propagative plant material, and considering that about 90% of economically important crops grown from seeds, seed pathology emerged as an important discipline. Seed health testing is presently part of quality analysis and carried out by seed enterprises and governmental institutions looking forward to exclude a new pathogen in a country or site. The development of seedborne pathogens detection methods has been following the plant pathogen detection and diagnosis advances, from the use of cultivation on semi-selective media, to antibodies and DNA-based techniques. Hyperspectral imaging (HSI) associated with artificial intelligence can be considered the new frontier for seedborne pathogen detection with high accuracy in discriminating infected from healthy seeds. The development of the process consists of standardization of methods and protocols with the validation of spectral signatures for presence and incidence of contamined seeds. Concurrently, epidemiological studies correlating this information with disease outbreaks would help in determining the acceptable thresholds of seed contamination. Despite the high costs of equipment and the necessity for interdisciplinary collaboration, it is anticipated that health seed certifying programs and seed suppliers will benefit from the adoption of HSI techniques in the near future.

## Introduction

Food security is currently the focus of agricultural studies in search for sustainable production solutions that keep up with population growth. It is estimated that by 2050 the world will have approximately 10 billion inhabitants (Godfray et al., 2010; Tilman et al., 2011), which makes the demand for food a current challenge. One of the most important limitation for food production are plant diseases which can cause significant losses in productivity and food quality around the world (Savary et al., 2019). Fungal and bacterial pathogens can reduce crop yields by around 15% and plant viruses by a range of 3% to 7%, under favorable conditions such microorganisms can cause losses of up to 100% (Oerke and Dehne, 2004).

Around 90% of crops of economic and food importance are grown by seeds (Maude, 1996), therefore, the seed health is fundamental for agricultural production. Seeds are a primary source of plant pathogen contamination in crop fields and specific tools for identification and early diagnosis of pathogens in seeds are essential to mediate disease management in crops and avoid losses caused by them (Gitaitis and Walcott, 2007; Dutta et al., 2014). Some requirements are desired in the selection of seed health diagnosis methods, such as specificity; sensitivity; efficiency/speed; simplicity; a satisfactory cost-effectiveness; and reliability (Marcinkowska, 2002).

The application of molecular and serological detection methods, despite presenting specificity and sensitivity, are destructive, mostly limited to laboratory application, requiring qualified labor (Kumar and Gupta, 2020). In addition, it does not make it possible to monitor the progress of the disease in the plant or even the action of compounds applied to the crop to control the spread of the pathogen (Mishra et al., 2020). The step forward for a more efficient detection of plant pathogens, focused on quantification and distribution would be the use of hyperspectral images, which could be associated with artificial intelligence, for a faster and more reliable streategy. This article is a state-of-the-art review on the detection of plant pathogens in seeds, emphasizing the recent advances with the use of hyperspectral images as a resource in the diagnosis of phytopathogens disseminated by seeds.

## Conventional methods for detecting plant pathogens in seeds

Initially, the detection of pathogens in seeds was carried out using visual diagnosis methods. Some pathogens, mainly fungi, cause external symptoms in seeds that can be naked-eye visualized or with the aid of a microscope (Gaur et al., 2020). The presence of certain symptoms in seeds may be common among plant pathogens or even the signs may not be visible in the seed tissues (Kumar et al., 2020). Furthermore, the symptoms in seeds may also be of abiotic origins, such as environmental stresses and nutrition to the seed crops or mechanical injuries during the seed harvesting and processing (Bala, 2020).

Directed detection by pathogen cultivation by plaquing the seed on semi-selective or enriched culture media is one of the most used techniques for detecting plant pathogens in seeds (Mancini et al., 2016). Although efficient, they are restricted to the detection of only cultivable microorganisms. After the pathogen growth, identification can be performed by morphology, biochemical and/or molecular tests (Kumar et al., 2020).

ELISA (Enzyme-linked immunosorbent assays), a sorologial method which demands species-specific antibodies in order not to cause cross-reactions between related and unrelated species which could lead to false negative results (Dewey et al., 1997; Mancini et al., 2016). Moreover, these tests often yield false positives by detecting non-viable microorganisms. This impacts analysis interpretation and directly affects diagnostic accuracy (Walcott, 2003; Afouda et al., 2009).

Molecular techniques, such as nucleic acid amplification using DNA and RNA, offer enhanced sensitivity and specificity in pathogen detection. PCR (Polymerase chain reaction), a pivotal advancement in molecular biology by Mullis et al. (1986), has evolved into various forms, including Nested PCR, BIO-PCR, qPCR (Quantitative Polymerase Chain Reaction), RT-qPCR (Reverse Transcription Quantitative Polymerase Chain Reaction), and LAMP (Loop-mediated isothermal amplification) (Massung et al., 1998; Notomi et al., 2000; Nazarenko et al., 2002; Bookout et al., 2006).

Conventional PCR is applied in several studies involving the detection of plant pathogens transmitted by seeds. However, these reproductive units are often infected by pathogens at a very low concentrations, which makes detection by PCR unfeasible due to insufficient amount of the target DNA. Based on this problem, BIO-PCR, proven to be effective for bacteria and fungi detection in seeds (Munkvold, 2009) as it allows the pathogen growth by incubation with semi-selective media followed by PCR (Schaad et al., 1995). Compared to conventional PCR, BIO-PCR is more advantageous due to the greater sensitivity, absence of PCR inhibitors (from plant or seed tissues), and detection of only viable cells, eliminating false positives (Marcinkowska, 2002). However, it may have higher costs due to the use of semi-selective media with expensive antibiotics, and delays from pathogen growth in the culture medium (Walcott, 2003; Schena et al., 2004).

Nested PCR is a very sensitive PCR modification, which can be completed in a 24-hour period (Chen et al., 2007). However, it demands additional time and reagents due the two rounds of PCR, and is more prone to contamination than conventional PCR (McCartney et al., 2003; Atkins and Clark, 2004). qPCR and RT-qPCR are other types of PCR which have been used for detecting seed-transmitted plant pathogens due to their sensitivity and speed. While effective, this technique may yield false positives from occasional spikes in background fluorescence or minimal DNA cross-contamination (Robène et al., 2015). Additionally, PCR-based analyses demand specialized and trained technical personnel and expensive equipment, usually being restricted to laboratory settings (Kaur et al., 2020). PCR techniques, including PCR, Nested-PCR, BIO-PCR, qPCR, RT-qPCR and others may be used as a multiplex, capable of detecting simultaneously more than one seed-borne pathogens with greater sensitivity than conventional and individual PCR (Ha et al., 2009; Robène-Soustrade et al., 2010).

Derived from PCR, Loop-mediated isothermal amplification (LAMP) based technique efficiently detects plant pathogens in seeds (Choudhary et al., 2022), offering several advantages such as: it operates under isothermal conditions, allows direct visualization of amplification results, and can be applied in the field (Notomi et al., 2000). The LAMP assay is more sensitive and specific than PCR, mainly due to the addition of loop primers (Nagamine et al., 2002). However, the handling of samples when adding interleaved dyes, which allow visualization of the result, can influence the occurrence of false positives, due to the release of aerosols of target nucleic acid fragments into the environment (Hardinge and Murray, 2019). Also, as other molecular tests, it may detect not only the living microrganisms but also any remaining pathogen DNA within the sample.

## Hyperspectral imaging and artificial intelligence

The human eye can detect only a small portion of the electromagnetic spectrum, distinguishing the spectral responses of images/objects in a restricted range of the visible spectrum between 400 nm and 700 nm (Zwinkels, 2015). To overcome this limitation, optical remote sensing has emerged as a technique capable of obtaining representative data in ranges beyond infrared, typically between 400 nm and 2500 nm (Oliveira et al., 2020). The possibility of capturing the spectral signal in a larger range allows the structuring of reflectance profiles and the detection of patterns that differentiate features between different targets far beyond the visual range (Shanmugapriya et al., 2019).

In recent decades, several studies have involved the application of remote sensing in agriculture both in field and laboratory. The use of optical remote sensing through hyperspectral images is an example (Demattê et al., 2010; Feng et al., 2019; Abdulridha et al., 2020b). These sensors measure the reflectance of an object, such as plant leaves, and identify any changes, such as those caused by a plant pathogen, as any disturbance to the leaf region would alter the reflectance and direction of the light (Xie et al., 2008).

A HSI (hyperspectral imaging) combines conventional imaging system with spectroscopy. Unlike the RGB (red, green and blue) color system, HSI corresponds to a set of techniques capable of capturing wavelengths beyond red, green and blue (Li et al., 2014). HSI consists of a spectrograph capable of capturing reflectance in a wide range of the spectrum, such as visible (VIS), ultraviolet (UV), near-infrared (NIR), and short-wave infrared (SWIR) (Bock et al., 2010). Such images are three-dimensional (3D), formed by two spatial dimensions (x and y) and one spectral dimension (z). By combining conventional imaging with spectroscopy, HSI obtains complementary information from both fields (Mishra et al., 2017). An example of such a combination is that while spectroscopy collects data about plant physiology, conventional imaging system gathers information on plant structural dynamics (Montes et al., 2007; Bucksch et al., 2014).

Hyperspectral images have hundreds of wavelength bands close together in a spectral range, producing a dense colorfull cube of information, with spatial resolution capable of obtaining several pixels per target (Lowe et al., 2017). The spectral portion generally applied to the study of hyperspectral images of plants ranges from UV starting at ~250 nm to SWIR up to ~2500 nm. The most useful spectral band for plant analysis are the visible and near infrared (Lowe et al., 2017). In the range of 400-700 nm, it is possible to capture changes in leaf pigmentation, whereas at 700-1300 nm range, changes in the cellular structure of the mesophyll. Wider spectral ranges (1300-2500 nm) are needed, for example, for water content in a plant (Peñuelas and Filella, 1998).

The greatest advantage of the application of HSI is that they are techniques capable of detecting and differentiating a disease even in an asymptomatic plant (Martinelli et al., 2015; Whetton et al., 2018). The possibility of early detection through the use of hyperspectral sensors can contribute to the early and efficient management of diseases, preventing spread in the field (Abdulridha et al., 2020a). Furthermore, hyperspectral imaging technology is non-invasive/destructive, which is advantageous when compared to molecular analysis, where the sample cannot be reused (Sankaran et al., 2010; Golhani et al., 2018).

The data generated is quite collinear and requires different statistical tools to extract information and model patterns (Mishra et al., 2017), such as for artificial intelligence. The application of artificial intelligence using machine learning and deep learning combined with hyperspectral images has been promising in several areas of agricultural management (Demattê et al., 2010; El-Mesery et al., 2019; Feng et al., 2019; Abdulridha et al., 2020b). Artificial intelligence has different definitions, succinctly, it refers to the ability of a machine to perform a function in a similar way to the human mind for “learning” and “problem solving”. Machine learning consists of computational algorithms that learn from input data and can perform classification or clustering tasks, which are ideal for finding patterns and trends in hyperspectral data (Dhakal et al., 2023).

Machine learning is a subfield of artificial intelligence and deep machine learning is a subset that integrates machine learning (Nguyen et al., 2019). Deep learning is excellent for analyzing and extracting useful data in large quantities or collected from different sources (Zhang et al., 2020). The analysis of hyperspectral images using deep learning with convolutional neural networks has emerged as a favorable methodology for evaluating and managing various conditions in agricultural crops, mainly aimed at disease diagnostics. Neural networks are designed to learn the spatial features that best classify or quantify a target/object, in the case of plant diseases, classifying and distinguishing, for example, healthy leaves from those infected by pathogens (Kattenborn et al., 2021).

In addition to deep learning, other algorithmic models based on machine learning are applied in hyperspectral data processing. Examples are: PCA (Principal Component Analysis) and LDA (Linear Discriminant Analysis) used for extracting features of interest, similarities, and dissimilarities in the data (Dua et al., 2020; Hsieh and Kiang, 2020), and SVM (Support Vector Machine), for solving linear classification problems, which are widely employed for HSI data classification due to their ability to effectively separate heterogeneous samples on the mapped plane (Kale et al., 2017). Although SVM was designed to decipher linear datasets, its usage can be extended to non-linear data when combined with kernel methods (Melgani and Bruzzone, 2004).

Segmentation, an important step before delivering the data cube to test a model, is applied for noise removal from the images, extraction of useful and non-redundant features and establishing the relationship between similar data points in the sample. Therefore, enabling the selection of regions of interest (ROIs), thereby allowing for more accurate data classification. However, segmentation requires the use of methods that demonstrate satisfactory performance for ROI selection. There are algorithms that can assist in this aspect, each with its own peculiarities. Furthermore, some studies clarify which algorithms exhibit better performance according to the type of HSI data to be addressed (Grewal et al., 2023). For seeds, few publications describe the issues associated to those plant material, as the variance of reflectance on each seed, due surface deformities and specular reflection, increasing the difficulties of segmentation (Barbedo et al., 2015).

Another importante step is the fusion of spectral and spatial data, which allows for the enhancement of spatial resolution in HSI images, while preserving spectral quality. However, the fusion process is complex, as there is typically a trade-off between preserving spatial data and spectral quality (Mookambiga and Gomathi, 2016). It is from this perspective that artificial intelligence has proven to be efficient in solving problems related to HSI data fusion, such as deep learning, which is ideal for extracting characteristics from complex and abundant data (Li et al., 2022). Additionally, there is commercial software available that can integrate these steps, simplifying the process.

## Detection of plant pathogens in seeds by hyperspectral imaging

The detection of plant pathogens in seeds using hyperspectral images associated with artificial intelligence mechanisms is described for different pathosystems (**Table 1**). A limited number of studies have been published specifically focusing on the detection of plant pathogens on seeds. The majority of these studies utilize prediction models for data processing, given that alterations on these organs may not be as visually discernible as those on fully grown plants, such as symptoms of necrosis or others. The low number of publications (22) and erratic distribution along the years reveals that it is still an open field to be explored, which probably concerns to the necessity of deep knowledge on the machine and algorithmic possibilities and their high costs.

**Table 1:** Compilation of publications focusing on the utilization of hyperspectral imaging for the detection of plant pathogens in seeds.

Seeds	Samples	Pathogen	Inoculation Method*	Reference Method	Spectral Range	Model Classification	Accuracy	Reference
Peanut	–	*Aspergillus* spp.	Naturally infected	Visually confirmed	970-2570 nm	PCA	98.73%	(Jiang et al., 2016)
Peanut	–	Diverse fungi species	Naturally infected, favorable conditions	Visually confirmed	967-2499 nm	SVM	>94%	(Qiao et al., 2017)
Peanut	600	*Aspergillus flavus*	Naturally infected, favorable conditions	Visually confirmed	400-1000 nm	CatBoost, GBDT, XGBoost, LightGBM	>97.42%	(Wu et al., 2022)
Rice	926	*Villosiclava virens*	Artifically inoculated/Naturally infected	PCR	874.41-1734.91 nm	PLS-DA, SVM, ELM	>94%	(Wu et al., 2020)
Rice	210	*Aspergillus oryzae*	Artifically inoculated	Visually confirmed	400-1000 nm	PLSR	–	(Siripatrawan and Makino, 2015)
Rice	47570	*Fusarium* spp.	Artifically inoculated	Visually confirmed	874.41 - 1734.91 nm	Convolution Neural Networks (CNNs), PLS-DA, SVM	>90%	(Wu et al., 2024)
Oat	–	*Fusarium* spp.	Naturally infected	Visually confirmed	1000-2500 nm	PLSR, PLS-LDA	–	(Tekle et al., 2015)
Canola	900	*Aspergillus glaucus*	Artifically inoculated	–	1000-1600 nm	LDA, QDA, MDA	>90%	(Senthilkumar et al., 2015)
Choy Sum	1630	*Penicillium decumbens*	Artifically inoculated	Visually confirmed	400-1000 nm	SMOTE-siPLS-stacking	>99%	(Xie et al., 2024)
Corn	892	*Aspergillus* spp.	Naturally infected	Culture media	935-1700 nm	PW-PCA-SVM	100,00%	(Chu et al., 2020)
Barley	3000	*Aspergillus glaucus, Penicillium* spp.	Artifically inoculated	–	1000-1600 nm	LDA, QDA, MDA	>80%	(Senthilkumar et al., 2016)
Pea, Bean, Chickpea, Lentil	1500	*Aspergillus flavus, Penicillium commune*	Artifically inoculated	–	900-1700 nm	LDA, QDA	>96%	(Karuppiah et al., 2016)
Watermelon	96	Cucumber green mottle mosaic virus	Naturally infected	RT-qPCR	948-2016 nm	PLS-DA	83.30%	(Lee et al., 2016a)
Watermelon	405	Cucumber green mottle mosaic virus	Naturally infected	RT-qPCR	950-2500 nm	LS-SVM	92%	(Seo et al., 2019)
Watermelon	336	*Acidovorax citrulli*	Artifically inoculated	Culture media	400-1000 nm	PLS-DA/LS-SVM	> 90%	(Lee et al., 2017b)
Watermelon	48	*Acidovorax citrulli*	Artifically inoculated	–	400-1800 nm	ANOVA	75%	(Lee et al., 2017a)
Corn	36	*Fusarium verticillioides*	Naturally infected	Visually confirmed	960-1662 nm	PLS-DA	77%	(Williams et al., 2010)
Wheat	120	*Fusarium* sp.	Naturally infected	Visually confirmed	1000-1700 nm	PLS-DA	100%	(Serranti et al., 2013)
Wheat	803	*Fusarium graminearum, Fusarium meridionale*	Naturally infected	Visually confirmed	528-1785 nm	LDA	91%	(Barbedo et al., 2015)
Wheat	21376	*Fusarium graminearum*	Naturally infected	Visually confirmed	938–1654 nm	LDA, PLS-DA	>92%	(Delwiche et al., 2019)
Wheat	1200	*Penicillium* spp., *Aspergillus glaucus*, *Aspergillus niger*	Artifically inoculated	–	1000-1600 nm	LDA, QDA, MDA	>95%	(Singh et al., 2007)
Wheat	800	*Fusarium graminearum*	Naturally infected	Visually confirmed	400-1000 nm	LDA	92%	(Shahin and Symons, 2011)

CNN (Convolutional neural network).

ELM (Extreme learning machine).

FDA (Factorial discriminant analysis).

LDA (Linear discriminant analysis).

LS-SVM (Least-squares support vector machines).

MDA (Multiple discriminant analysis).

PCA (Principal component analysis).

PLS-DA (Partial least squares-discriminant analysis).

PLSR (Partial least squares regression).

QDA (Qualitative data analysis).

SVM (Support vector machine).

SVR (Support vector regression).

“-” Information not provided.

*Naturally infected seeds also includes inoculated plants at flower of fruit development stages.

**Figure 1:**
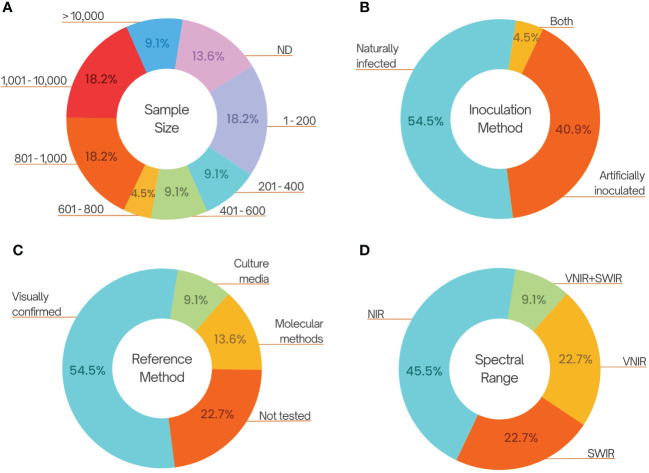
Compilation of cited publications on hyperspectral imaging for the detection of plant pathogens in seeds. **(A)** Sample size used in each publication. **(B)** Method of inoculation for the production of infested/infect seeds. **(C)** Used methods for the confirmation or quantification of the pathogen within seeds. **(D)** The intervals of wavelenght used for the hyperspectral image production, 400-1000 (VNIR), 700-2500 nm (NIR), 1000-2500 nm (SWIR).

The variables tested in these studies include sample size, the method for producing infected seed samples, the reference method for confirming the infection, as well as the wavelengths applied and algorithms utilized. The number of sample size among the publications is variable, from not informing the number of seeds applied in the study up to 47 thousand seeds (**Figure 1A**). The low number of seeds used to train and test the algorithm can superestimate the accuracy. Several publications utilize artificially inoculated seeds (40,9%), often through the infiltration of propagule suspensions, as positive controls for image generation (**Figure 1B**), while 54,5% used naturally infected seeds, including those for the detection of mycotoxin-producing fungi. Artificially inoculated or infiltrated seeds are commonly utilized to generate contaminated seed lots. However, this approach may introduce potential inaccuracies in the data, as the concentration of the pathogen could surpass levels typically observed in natural conditions. Wu et al. (2020) states that laboratory-inoculated seeds reflects accurately the detection of field-infected kernels. Nevertheless, such inoculated seeds might not express the impact of infection on the physiology, chemistry, and other compositions of the seed, which could be detected by hyperspectral imaging (HSI), as it can happen to soy seeds infected with *Fusarium verticillioides*, which may change flavonoids content (Pedrozo and Little, 2017). Further studies are required to validate this data. Additionally, the knowledge regarding the threshold limit of propagules on a seed for disease transmission, from germination to seedlings, potentially resulting in disease outbreaks, is crucial. This knowledge impairs the use of the method in properly detecting potentially harmful infected seeds (those that lead to disease transmission). For reference method, which is a method for confirmation of the pathogen presence within the seed, some publications (22.77%) did not use any kind of evaluation, while 54,5% assessed it visually (**Figure 1C**) and three publications (13,6%) used molecular methods. Concerning the wavelenghs applied, VIS, NIR, VNIR, SWIR bands (ranging from 400 to 2500 nm) were used in several studies, with a predominancy of NIR, occasionally in combination (**Figure 1D**).

Most studies focused on the detection of fungi, particularly those associated with the production of mycotoxins, such as *Fusarium* and *Aspergillus* species (**Table 1**). Mycotoxin accumulation renders grains unfit for human and animal consumption. Few studies address the dispersion of fungi by seeds, which could introduce the pathogen and/or cause an outbreak on production fields. Notably, groups of pathogens such as bacteria and viruses received limited attention, despite the fact that several important diseases transmitted by seeds fall within these categories. In the case of bacterial pathogens, Lee et al. (2017b) applied hyperspectral imaging in the near-infrared range with two models to classify healthy watermelon seeds and those infected with *Acidovorax avenae* subsp. *citrulli*. The accuracy of both PLS-DA and LS-SVM models was of 91.7% and 90.5%, respectively. Using the Raman hyperspectral imaging technique, they achieved 75% of classification accuracy (Lee et al., 2017a). On its turn, for viruses, using the PLS-DA model, Lee et al. (2016a) distinguished watermelon seeds infected by Cucumber green mottle mosaic virus (CGMMV) with an accuracy of 83.3% while Seo et al. (2019) obtained 92% accuracy using the LS-SVM (Least squares support vector machine) model.

For plant pathogenic fungi, numerous studies have investigated seed detection using hyperspectral images. Notably, some have demonstrated high accuracy in classification. For instance, Chu et al. (2020) achieved 100% accuracy using PCA and SVM models to classify hyperspectral data from both healthy and *Aspergillus* spp. infected corn seeds. Wu et al. (2022) utilized hyperspectral images in the NIR range to identify peanut seeds naturally infected with *Aspergillus flavus*, reaching an accuracy exceeding 97% with three distinct classification models. In Wu et al. (2024) study, the combination of hyperspectral data acquisition and sample classification using convolutional neural networks yielded an accuracy of over 98% in identifying rice seeds infected with *Fusarium* spp. Additionally, employing other learning models like PLS-DA and SVM resulted in accuracies surpassing 90%.

The detection of nematodes in seeds involving hyperspectral is still an open area, as no publication has been found, probably due to the fact that most plant nematode are transmited by vegetative materials, such as potato tubers, with few species capable of infecting botanic seeds. Žibrat et al. (2021) and Lapajne et al. (2022) evaluated the use of hyperspectral imaging and the application of different algorithmic models and obtained an accuracy of around 100% in the classification of infected seed potato tubers with *Meloidogyne luci*.

## Final comments and perspectives

Due to its non-destructive nature, hyperspectral imaging might be applied to 100% of seeds without the need for sampling or destruction, as the case of molecular methods. Moreover, the utilization of hyperspectral imaging for detection does not preclude the use of molecular diagnosis as shown by Wu et al., 2020; instead, the integration of these methods enhances reliability and specificity in pathogen detection. The application of hyperspectral imaging diagnostics in seeds can expedite the detection of microorganisms, thereby lowering the chances of the introduction of new plant pathogens or variants in a site. However, much remains to be studied in order to enhance the reliability of this technology, aligning it with the standards observed in other scientific domains that already uses hyperspectral imaging.

Several difficulties in this area serve as deterrents for the adoption of this technology, including the high costs of equipment, complex software and methods. Furthermore, the involvement of a multidisciplinary team including plant pathologists, along with electronic engineers, physicists, and software engineers, is strongly recommended to deal with this new research frontier. Unfortunately, it can be observed in the recent published publications that plant pathologists are not present as part of their research teams. With the knowledge of epidemics, agricultural producing systems and seed-borne pathogen infection and detection, such especialist could shorten the period for technology application and adoption by the producing chaim authors.

Concerning the generation of contaminated seed lots for the studies, prior to the development of the hyperspectral imaging protocol, assays should be carried out to simulate the most natural conditions of an infected seed. Alternatively, naturally infected seeds obtained through either natural or artificial infection of plants could be utilized. However, this alternative approach introduces a different challenge: determining the method to validate the presence of the plant pathogen on a specific seed. Another issue is the lack of information regarding the spectral signature of the putative alterations caused to the seed by the presence of the pathogen and its successful transmission to the plant. Besides, sample standards need to be defined for each crop species.

**Figure 2:**
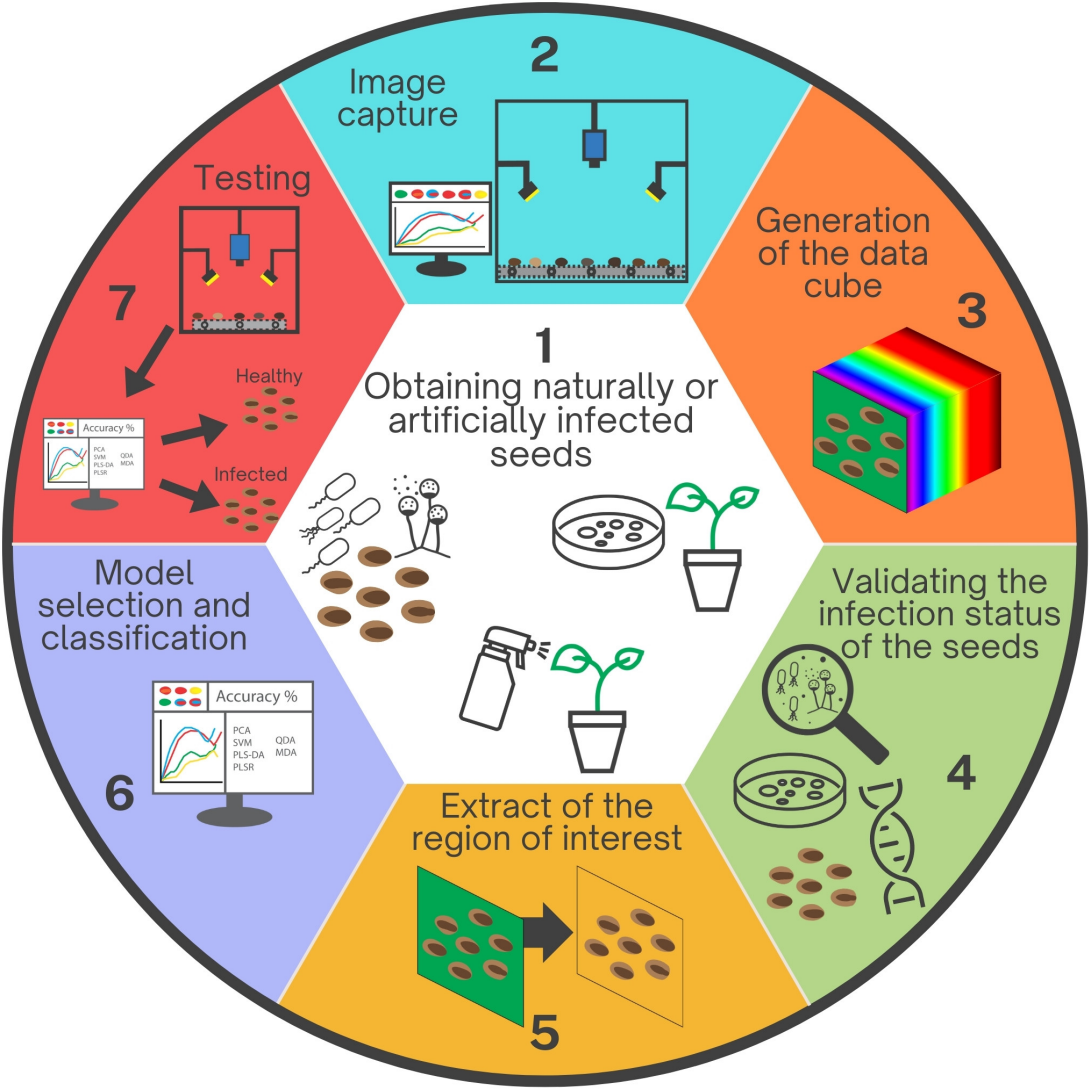
Sequential stages involved in developing hyperspectral imaging for detecting plant pathogens within seeds. 1. Production or acquisition of seeds naturally or artificially infected by inoculating seeds, flowers, or fruits. 2. Image capture utilizing a hyperspectral camera. 3. Data cube generation by integrating multiple wavelength readings. 4. Validation of the infection status of each seed through various detection methods, such as culture media or molecular techniques. 5. Background removal and selection of the region of interest. 6. Selection of machine learning/deep learning models based on the seeds identified in step 4, taking into account the accuracy of each model. 7. Testing the developed protocol and algorithm to validate the technique efficacy.

Despite these aspects, the analyzed publications collectively highlighted important approaches that should be considered for the establishment of protocols for hyperspectral imaging detection technologies. Therefore, a seven-step scheme is proposed and recommended here as a template for future studies (**Figure 2**): obtention of contaminated seeds, preferably naturally infected; images capture; the data cube generation; assessment of the infection status of each seed; segmentation or extraction of the region of interest; model selection and classification based on sample size and accuracy; and finally, testing with a larger sample.

Hyperspectral imaging studies for seed detection are relatively recent, but they hold significant potential for the development of new protocols, particularly in the context of the prevailing era of robotics and artificial intelligence. The adoption of hyperspectral imaging detection technologies stands to offer substantial advantages in the production of healthy seeds, particularly in terms of enhanced reliability and practicality. This technology holds great promise for certification authorities and has the potential to bolster the confidence of farmers in this agricultural inputs, if a substantial amount of them can be checked for the presence of important seed-borne pathogens.

## Author contributions

LF: Data curation, Writing – original draft. IC: Writing – review & editing. LJ: Writing – review & editing. AQ: Writing – review & editing. MR: Conceptualization, Funding acquisition, Supervision, Writing – review & editing.
